# Supplementary material for: Machine Learning Accelerates Crystallization for Structure Determination
Source: Angew Chem Int Ed Engl. 2026 May 4;65(25):e1218503. doi: 10.1002/anie.1218503 (PMC13266958; doi:10.1002/anie.1218503)

checkCIF/PLATON report

Structure factors have been supplied for datablock(s) lcz101

THIS REPORT IS FOR GUIDANCE ONLY. IF USED AS PART OF A REVIEW PROCEDURE FOR PUBLICATION, IT SHOULD NOT REPLACE THE EXPERTISE OF AN EXPERIENCED CRYSTALLOGRAPHIC REFEREE.

No syntax errors found.   CIF dictionary   Interpreting this report

Datablock: lcz101

Bond precision:	C-C = 0.0115 A		Wavelength=1.54184
Cell:	a=21.6879(2)	b=24.5290(2)	c=25.4511(2)
	alpha=90	beta=91.009(1)	gamma=90
Temperature:	100 K		

	Calculated	Reported
Volume	13537.4(2)	13537.4(2)
Space group	P 21/n	P 1 21/n 1
Hall group	-P 2yn	-P 2yn
Moiety formula	C15 H3 Ag3 F18 N6, C13 H12	4(C15 H3 Ag3 F18 N6), 4(C13 H12)
Sum formula	C28 H15 Ag3 F18 N6	C112 H60 Ag12 F72 N24
Mr	1101.07	4404.28
Dx,g cm-3	2.161	2.161
Z	16	4
Mu (mm-1)	15.026	15.025
F000	8448.0	8448.0
F000’	8488.20	
h,k,lmax	27,30,31	27,30,31
Nref	27877	26482
Tmin,Tmax	0.144,0.178	0.253,1.000
Tmin’	0.060	

Correction method= # Reported T Limits: Tmin=0.253 Tmax=1.000
AbsCorr = MULTI-SCAN

Data completeness= 0.950 Theta(max)= 74.967

R(reflections)= 0.0487(22360) wR2(reflections)= 0.1332(26482)

S = 1.035 Npar= 1985

The following ALERTS were generated. Each ALERT has the format
test-name_ALERT_alert-type_alert-level.
Click on the hyperlinks for more details of the test.

Alert level C

```
PLAT041_ALERT_1_C Calc. and Reported SumFormula    Strings  Differ     Please Check
              Calc: C28 H15 Ag3 F18 N6
              Rep.: C112 H60 Ag12 F72 N24
PLAT042_ALERT_1_C Calc. and Reported MoietyFormula Strings  Differ     Please Check
              Calc: C15 H3 Ag3 F18 N6, C13 H12
              Rep.: 4(C15 H3 Ag3 F18 N6), 4(C13 H12)
PLAT213_ALERT_2_C Atom F01N            has ADP max/min Ratio .....        3.1 prolat
PLAT213_ALERT_2_C Atom F064            has ADP max/min Ratio .....        3.4 prolat
PLAT213_ALERT_2_C Atom F02J            has ADP max/min Ratio .....        3.3 prolat
PLAT213_ALERT_2_C Atom F03N            has ADP max/min Ratio .....        3.3 prolat
PLAT213_ALERT_2_C Atom F02O            has ADP max/min Ratio .....        3.6 prolat
PLAT213_ALERT_2_C Atom F03V            has ADP max/min Ratio .....        3.2 prolat
PLAT213_ALERT_2_C Atom F044            has ADP max/min Ratio .....        3.4 prolat
PLAT213_ALERT_2_C Atom F05K            has ADP max/min Ratio .....        3.4 prolat
PLAT213_ALERT_2_C Atom F05P            has ADP max/min Ratio .....        3.3 prolat
PLAT213_ALERT_2_C Atom C05R            has ADP max/min Ratio .....        3.5 prolat
PLAT213_ALERT_2_C Atom C05Y            has ADP max/min Ratio .....        3.3 prolat
PLAT213_ALERT_2_C Atom C062            has ADP max/min Ratio .....        3.1 prolat
PLAT220_ALERT_2_C NonSolvent   Resd 1  F   Ueq(max)/Ueq(min) Range        4.3 Ratio
PLAT220_ALERT_2_C NonSolvent   Resd 4  F   Ueq(max)/Ueq(min) Range        3.5 Ratio
PLAT234_ALERT_4_C Large Hirshfeld Difference F05Z     --C061     .       0.23 Ang.
PLAT234_ALERT_4_C Large Hirshfeld Difference F063     --C061     .       0.16 Ang.
PLAT234_ALERT_4_C Large Hirshfeld Difference C04S     --C061     .       0.16 Ang.
PLAT234_ALERT_4_C Large Hirshfeld Difference F02G     --C05S     .       0.16 Ang.
PLAT234_ALERT_4_C Large Hirshfeld Difference F05K     --C062     .       0.16 Ang.
PLAT234_ALERT_4_C Large Hirshfeld Difference C05R     --C05Y     .       0.17 Ang.
PLAT234_ALERT_4_C Large Hirshfeld Difference C05X     --C05Y     .       0.17 Ang.
PLAT241_ALERT_2_C High   'MainMol' Ueq as Compared to Neighbors of       C05Y Check
PLAT242_ALERT_2_C Low    'MainMol' Ueq as Compared to Neighbors of       C02P Check
PLAT250_ALERT_2_C Large U3/U1 Ratio for <U(i,j)> Tensor(Resd    7)        2.5 Note
PLAT342_ALERT_3_C Low Bond Precision on  C-C Bonds ..............      0.0115 Ang.
PLAT906_ALERT_3_C Large K Value in the Analysis of Variance ......      2.962 Check
PLAT911_ALERT_3_C Missing FCF Refl Between Thmin & STh/L=    0.600        179 Report
                7 28  0,   8 28  0,   3 22  1, -19 20  2, -12 21  2,   4 23  2,
                5 24  3,   1  6  5,   4 28  5, -19 19  6,   3 28  6,   4 28  6,
                5 28  6,  -1 14  7,  -8 27  7,   4 28  7,   5 28  7,  -7  8 11,
              -19 17 11,  17  2 12,  -8  6 12, -19 16 12,  13 21 13, -21  4 17,
               -6 22 19, -19  0 21, -19  1 21, -19  3 21,   6 12 21,   4 20 21,
                   (  149 More Missing: see the .ckf listing file)
PLAT934_ALERT_3_C Number of (Iobs-Icalc)/Sigma(W) > 10 Outliers ..          1 Check
              -12  0 22,
PLAT971_ALERT_2_C Check Calcd Resid. Dens.  0.98Ang From F064            2.07 eA-3
```

PLAT971_ALERT_2_C Check Calcd Resid. Dens. 1.17Ang From F064 2.05 eA-3

Alert level G

PLAT003_ALERT_2_G Number of Uiso or U(i,j) Restrained non-H-Atoms 5 Report
PLAT045_ALERT_1_G Calculated and Reported Z Differ by a Factor ... 4 Check
PLAT083_ALERT_2_G SHELXL Second Parameter in WGHT Unusually Large 63.29 Why ?
PLAT142_ALERT_4_G s.u. on b - Axis Small or Missing .... 0.00020 Ang.
PLAT143_ALERT_4_G s.u. on c - Axis Small or Missing .... 0.00020 Ang.
PLAT177_ALERT_4_G The CIF-Embedded .res File Contains DELU Records 2 Report
PLAT186_ALERT_4_G The CIF-Embedded .res File Contains ISOR Records 1 Report
PLAT192_ALERT_3_G A Non-default DELU Restraint Value for SecondPar 0.0200 Report
PLAT192_ALERT_3_G A Non-default DELU Restraint Value for SecondPar 0.0200 Report
PLAT242_ALERT_2_G Low 'MainMol' Ueq as Compared to Neighbors of C02Q Check
PLAT242_ALERT_2_G Low 'MainMol' Ueq as Compared to Neighbors of C033 Check
PLAT242_ALERT_2_G Low 'MainMol' Ueq as Compared to Neighbors of C03W Check
PLAT242_ALERT_2_G Low 'MainMol' Ueq as Compared to Neighbors of C04I Check
PLAT242_ALERT_2_G Low 'MainMol' Ueq as Compared to Neighbors of C04U Check
PLAT242_ALERT_2_G Low 'MainMol' Ueq as Compared to Neighbors of C061 Check
PLAT242_ALERT_2_G Low 'MainMol' Ueq as Compared to Neighbors of C03C Check
PLAT242_ALERT_2_G Low 'MainMol' Ueq as Compared to Neighbors of C041 Check
PLAT242_ALERT_2_G Low 'MainMol' Ueq as Compared to Neighbors of C04V Check
PLAT242_ALERT_2_G Low 'MainMol' Ueq as Compared to Neighbors of C05B Check
PLAT242_ALERT_2_G Low 'MainMol' Ueq as Compared to Neighbors of C05F Check
PLAT242_ALERT_2_G Low 'MainMol' Ueq as Compared to Neighbors of C05U Check
PLAT242_ALERT_2_G Low 'MainMol' Ueq as Compared to Neighbors of C03O Check
PLAT242_ALERT_2_G Low 'MainMol' Ueq as Compared to Neighbors of C046 Check
PLAT242_ALERT_2_G Low 'MainMol' Ueq as Compared to Neighbors of C04X Check
PLAT242_ALERT_2_G Low 'MainMol' Ueq as Compared to Neighbors of C057 Check
PLAT242_ALERT_2_G Low 'MainMol' Ueq as Compared to Neighbors of C059 Check
PLAT242_ALERT_2_G Low 'MainMol' Ueq as Compared to Neighbors of C05O Check
PLAT242_ALERT_2_G Low 'MainMol' Ueq as Compared to Neighbors of C03G Check
PLAT242_ALERT_2_G Low 'MainMol' Ueq as Compared to Neighbors of C058 Check
PLAT242_ALERT_2_G Low 'MainMol' Ueq as Compared to Neighbors of C05G Check
PLAT242_ALERT_2_G Low 'MainMol' Ueq as Compared to Neighbors of C05N Check
PLAT242_ALERT_2_G Low 'MainMol' Ueq as Compared to Neighbors of C05S Check
PLAT242_ALERT_2_G Low 'MainMol' Ueq as Compared to Neighbors of C062 Check
PLAT434_ALERT_2_G Short Inter HL..HL Contact F01W ..F044 . 2.80 Ang.
1/2-x,-1/2+y,3/2-z = 2_546 Check
PLAT434_ALERT_2_G Short Inter HL..HL Contact F01W ..F02R . 2.82 Ang.
x,y,z = 1_555 Check
PLAT434_ALERT_2_G Short Inter HL..HL Contact F02A ..F05Z . 2.81 Ang.
-1+x,y,z = 1_455 Check
PLAT720_ALERT_4_G Number of Unusual/Non-Standard Labels .... 280 Note

Ag01	Ag02	Ag03	Ag04	Ag05	Ag06	Ag07	Ag08
Ag09	Ag0A	Ag0B	Ag0C	F00D	F00E	F00F	F00G
F00H	F00I	F00J	F00K	F00L	F00M	F00N	F00O
F00P	F00Q	F00R	F00S	N00T	F00U	F00V	N00W
N00X	F00Y	F00Z	F010	F011	F012	N013	N014
N015	N016	N017	F018	N019	N01A	N01B	N01C
F01D	N01E	F01F	N01G	F01H	F01I	N01J	F01K
F01L	F01M	F01N	F01O	F01P	F01Q	N01R	N01S
F01T	N01U	F01V	F01W	N01X	F01Y	N01Z	N020
N021	F022	F023	N024	F025	F026	F027	N028
F029	F02A	F02B	F02C	F02D	F02E	F02F	F02G
F02H	F02I	F02J	F02K	F02L	F02M	F02N	F02O
C02P	C02Q	F02R	C02S	C02T	C02U	H02U	C02V

```
              H02V    F02W    C02X    H02X    C02Y    C02Z    C030    F031
              C032    C033    C034    H034    C035    H035    C036    C037
              H037    F038    C039    C03A    H03A    C03B    H03B    C03C
              C03D    C03E    C03F    C03G    C03H    H03H    C03I    H03I
              C03J    C03K    H03K    C03L    H03L    C03M    F03N    C03O
              C03P    H03P    C03Q    H03Q    C03R    C03S    C03T    C03U
              F03V    C03W    C03X    H03X    C03Y    H03Y    C03Z    C040
              H040    C041    C042    C043    H043    F044    C045    C046
              C047    C048    C049    H049    C04A    H04A    C04B    H04B
              C04C    C04D    H04D    C04E    C04F    H04F    C04G    H04G
              C04H    C04I    C04J    C04K    H04K    C04L    C04M    C04N
              H04N    C04O    H04O    C04P    H04P    C04Q    H04Q    C04R
              H04R    C04S    C04T    H04T    C04U    C04V    C04W    C04X
              C04Y    H04Y    C04Z    H04Z    C050    H050    C051    C052
              H052    C053    H053    C054    H054    C055    H055    C056
              H056    C057    C058    C059    C05A    H05A    C05B    C05C
              H05J    C05D    H05D    C05E    H05E    C05F    C05G    C05H
              H05K    C05I    H05M    H05N    H05O    C05J    H05G    H05H
              H05I    F05K    C05L    H05L    C05M    C05N    C05O    F05P
              C05Q    H05Q    C05R    C05S    C05T    H05T    C05U    C05V
              H05B    H05C    H05F    C05W    C05X    C05Y    H05Y    F05Z
              C061    C062    F063    F064    C065    H06A    H06B    H06C
PLAT860_ALERT_3_G Number of Least-Squares Restraints ............         32 Note
PLAT883_ALERT_1_G Absent Datum for _atom_sites_solution_primary ..     Please Do !
PLAT912_ALERT_4_G Missing # of FCF Reflections Above STh/L=  0.600       1212 Note
PLAT941_ALERT_3_G Average HKL Measurement Multiplicity ..........         3.8 Low
PLAT969_ALERT_5_G The 'Henn et al.' R-Factor-gap value ..........      3.152 Note
              Predicted wR2: Based on SigI**2  4.23 or SHELX Weight 12.88
PLAT978_ALERT_2_G Number C-C Bonds with Positive Residual Density.          0 Info
```

---

   0 **ALERT level A** = Most likely a serious problem - resolve or explain
   0 **ALERT level B** = A potentially serious problem, consider carefully
  32 **ALERT level C** = Check. Ensure it is not caused by an omission or oversight
  43 **ALERT level G** = General information/check it is not something unexpected

   4 ALERT type 1 CIF construction/syntax error, inconsistent or missing data
  49 ALERT type 2 Indicator that the structure model may be wrong or deficient
   8 ALERT type 3 Indicator that the structure quality may be low
  13 ALERT type 4 Improvement, methodology, query or suggestion
   1 ALERT type 5 Informative message, check

Validation response form

Please find below a validation response form (VRF) that can be filled in and pasted into your CIF.

```
# start Validation Reply Form
_vrf_PLAT041_lcz101
;
PROBLEM: Calc. and Reported SumFormula    Strings  Differ     Please Check
RESPONSE: ...
;
_vrf_PLAT042_lcz101
;
PROBLEM: Calc. and Reported MoietyFormula Strings  Differ     Please Check
RESPONSE: ...
```

```
;
_vrf_PLAT213_lcz101
;
PROBLEM: Atom F01N            has ADP max/min Ratio .....        3.1 prolat
RESPONSE: ...
;
_vrf_PLAT220_lcz101
;
PROBLEM: NonSolvent   Resd 1  F   Ueq(max)/Ueq(min) Range        4.3 Ratio
RESPONSE: ...
;
_vrf_PLAT234_lcz101
;
PROBLEM: Large Hirshfeld Difference F05Z     --C061     .       0.23 Ang.
RESPONSE: ...
;
_vrf_PLAT241_lcz101
;
PROBLEM: High   'MainMol' Ueq as Compared to Neighbors of       C05Y Check
RESPONSE: ...
;
_vrf_PLAT242_lcz101
;
PROBLEM: Low    'MainMol' Ueq as Compared to Neighbors of       C02P Check
RESPONSE: ...
;
_vrf_PLAT250_lcz101
;
PROBLEM: Large U3/U1 Ratio for <U(i,j)> Tensor(Resd    7)        2.5 Note
RESPONSE: ...
;
_vrf_PLAT342_lcz101
;
PROBLEM: Low Bond Precision on  C-C Bonds ..............     0.0115 Ang.
RESPONSE: ...
;
_vrf_PLAT906_lcz101
;
PROBLEM: Large K Value in the Analysis of Variance ......      2.962 Check
RESPONSE: ...
;
_vrf_PLAT911_lcz101
;
PROBLEM: Missing FCF Refl Between Thmin & STh/L=    0.600        179 Report
RESPONSE: ...
;
_vrf_PLAT934_lcz101
;
PROBLEM: Number of (Iobs-Icalc)/Sigma(W) > 10 Outliers ..          1 Check
RESPONSE: ...
;
_vrf_PLAT971_lcz101
;
PROBLEM: Check Calcd Resid. Dens.  0.98Ang From F064            2.07 eA-3
RESPONSE: ...
;
# end Validation Reply Form
```

It is advisable to attempt to resolve as many as possible of the alerts in all categories. Often the minor alerts point to easily fixed oversights, errors and omissions in your CIF or refinement strategy, so attention to these fine details can be worthwhile. It is up to the individual to critically assess their own results and, if necessary, seek expert advice.

PLATON version of 17/09/2025; check.def file version of 17/09/2025

duplicate check

No duplication found

Datablock lcz101 - ellipsoid plot

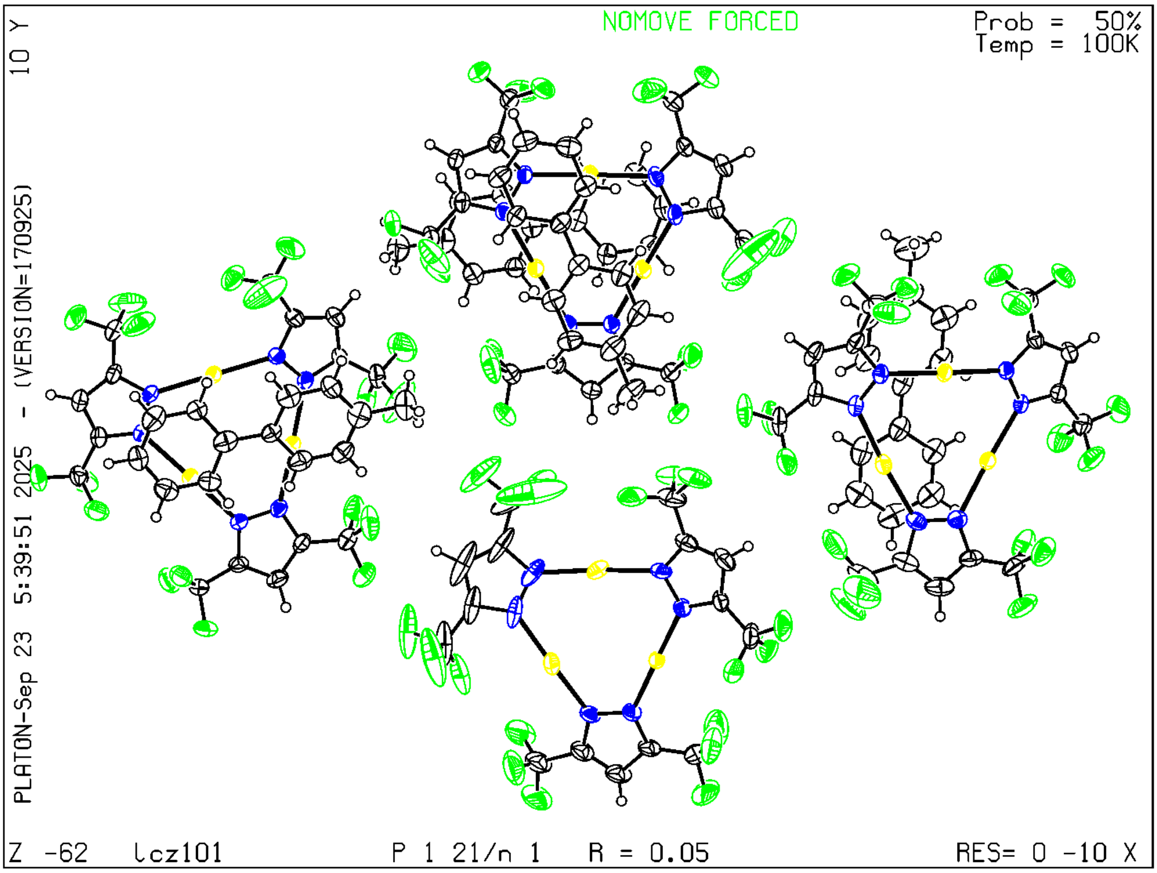

Supplement: Supplementary file 2 — Supporting File 2: anie72462‐sup‐0002‐DataFile.zip. [file ANIE-65-e1218503-s002.zip › supplementary_materials_crystallographic_data submit.zip!/cif_and_checkcif(1-114)/Ag3Pz3_101/Ag3Pz3_101_checkcif.pdf]
